# gwasrapidd: an R package to query, download and wrangle GWAS catalog data

**DOI:** 10.1093/bioinformatics/btz605

**Published:** 2019-08-02

**Authors:** Ramiro Magno, Ana-Teresa Maia

**Affiliations:** Centre for Biomedical Research (CBMR); Algarve Biomedical Center (ABC); Centre for Biomedical Research (CBMR); Algarve Biomedical Center (ABC); Department of Biomedical Sciences and Medicine (DCBM), Universidade do Algarve, Faro 8005-139, Portugal

## Abstract

**Motivation:**

The National Human Genome Research Institute Catalog of Published Genome-Wide Association Studies (GWAS) Catalog has collected, curated and made available data from over 7100 studies. The recently developed GWAS Catalog representational state transfer (REST) application programming interface (API) is the only method allowing programmatic access to this resource.

**Results:**

Here, we describe *gwasrapidd*, an R package that provides the first client interface to the GWAS Catalog REST API, representing an important software counterpart to the server-side component. *gwasrapidd* enables users to quickly retrieve, filter and integrate data with comprehensive bioinformatics analysis tools, which is particularly critical for those looking into functional characterization of risk loci.

**Availability and implementation:**

*gwasrapidd* is freely available under an MIT License, and can be accessed from https://github.com/ramiromagno/gwasrapidd.

**Supplementary information:**

Supplementary data are available at *Bioinformatics* online.

## 1 Introduction

The National Human Genome Research Institute (NHGRI) Catalog of published GWAS Catalog, created in 2014, is a publicly available, manually curated, database of all published genome-wide association studies (GWAS) (Welter *et al.*, 2014). Its latest data release [date July 12, 2019] includes data from 4054 publications and 143 963 unique SNP-trait associations for human diseases. Currently, these data can be accessed by three methods: (i) via the web graphical user interface (GUI), (ii) by downloading database dumps, or, more recently, (iii) via the GWAS catalog representational state transfer (REST) application programming interface (API), which provides direct programmatic access and hence is the preferred method for bioinformatics analyses.

We developed the first R package (R Core Team, 2017) allowing programmatic access to the GWAS catalog REST API: *gwasrapidd*. This package provides a simple interface for querying catalog data, abstracting away the informatic details of the REST API. In addition, retrieved data are mapped to in-memory relational databases of tidy data tables, allowing prompt integration with tidy-verse packages for subsequent transformation, visualization and modelling of data (Wickham *et al.*, 2014; Wickham and Grolemund, 2017).

## 2 Results

### 2.1 Retrieving data from the GWAS Catalog REST API

The GWAS Catalog REST API is an EBI service hosted at https://www.ebi.ac.uk/gwas/rest/api/. The REST API uses hypermedia with resource responses following the JSON hypertext application language (HAL) format (Kelly, 2016). Response data are, therefore, provided as hierarchical data in JSON format, can be paginated (i.e. split into multiple responses) and can also be embedded (i.e. have other resources contained within them), adding extra complexity to the returned JSON format [Additional File 1: Supplementary Table S1, and (NHGRI-EBI GWAS Catalog Team, 2019)].

**Fig. 1.**
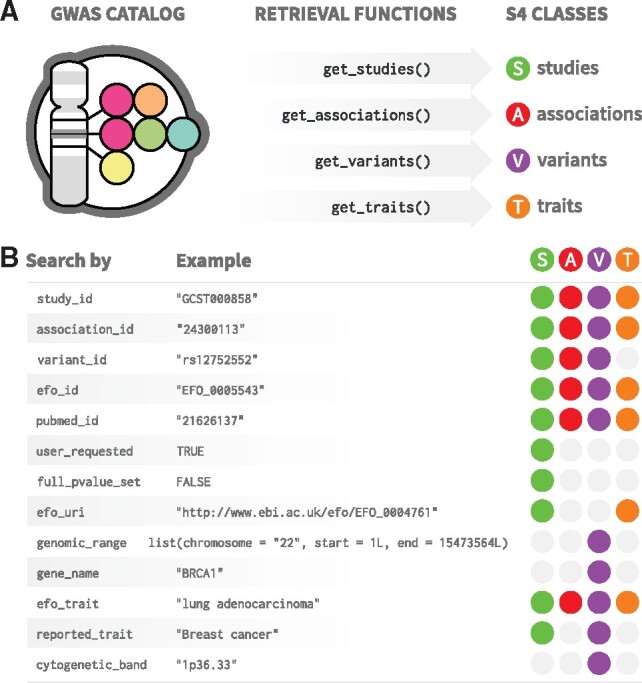
*gwasrapidd* retrieval functions. **(A)** Functions for retrieving data from the GWAS Catalog: get_studies(), get_associations(), get_variants() and get_traits(). **(B)** *gwasrapidd* search criteria (function parameters) to be used with retrieval functions. Colored circles indicate which entities can be retrieved by which criteria

To ease the conversion from the hierarchical to the relational tabular format—the preferred format for data analysis in R (Wickham and Grolemund, 2017), and to abstract away the informatic details associated with the HAL format, we developed a set of retrieval functions (Fig. 1A). Since the REST API data are organized around four core data entities —*studies*, *associations*, *variants* and *traits* (NHGRI-EBI GWAS Catalog Team, 2019)— we implemented four corresponding retrieval functions that encapsulate the technical aspects of resource querying and format conversion: get_studies(), get_associations(), get_variants() and get_traits() (Fig. 1A). These functions simplify the querying of GWAS entities, by providing a complete and consistent interface to the Catalog. For example, to query for *studies*, the user needs only to know the function get_studies(), whereas the REST API itself exposes a set of disparate resource URL endpoints for *studies* following the available search criteria [Additional file 1: Supplementary Table S1, and (NHGRI-EBI GWAS Catalog Team, 2019)]. Moreover, the user can choose from any number of available search criteria exposed by the REST API directly as arguments to the retrieval functions (Fig. 1B). All arguments are vectorized, meaning that multiple queries are promptly available from a single function call. Results obtained from multiple queries can be combined in an OR or AND fashion with the set_operation parameter. If set_operation is set to OR (default behavior), results are collated while removing duplicates, if any. If set_operation is set to AND, only entities that concomitantly match all criteria are returned. If finer control is needed on combining results, the following functions can be used: bind(), union(), intersect(), setdiff() and setequal(). These are S4 methods that work with the S4 classes created in *gwasrapidd* (Additional File 2: Figure S1). An example of a case study can be found in Additional File 3.

### 2.2 Representation of GWAS Catalog entities

All S4 classes share the same design principles that makes them relational databases: (i) each slot corresponds to a table (data frame in R), (ii) the first slot corresponds to the main table that lists observations of the respective GWAS Catalog entity, e.g. *studies* and, (iii) all tables have a primary key, the identifier of the respective GWAS Catalog entity: study_id, association_id, variant_id or efo_id (Additional File 2: Supplementary Fig. S1). For easy consultation of the variables in the tables, we provide a cheat-sheet (Additional File 4: *gwasrapidd* cheat-sheet); for the detailed description the user can issue the following commands to open the help page about each class: class?studies, class?associations, class?variants or class?traits.

### 2.3 Improvements and limitations

Compared to the exposed REST API, we have augmented the search possibilities in *gwasrapidd* in two ways: (i) by allowing searches for *variants* by cytogenetic region (as is possible with the web GUI) and (ii) by allowing searching *variants* by EFO identifier (efo_id), indirectly via EFO traits get_traits(). The first was implemented by embedding a dataset of genomic ranges of the human cytogenetic bands in *gwasrapidd*, so that queries made by cytogenetic band can be translated into searches by genomic range (genomic_range). Additionally, *gwasrapidd* also provides a set of helper functions to easily browse linked web resources, such as PubMed [open_in_pubmed()], dbSNP [open_in_dbsnp()] and GTEx project [open_in_gtex()].

Currently, the limitations of the REST API when compared to the web GUI are: (i) it is not possible to perform free text searches, and (ii) it is not possible to search traits using child trait terms automatically, they need to be included explicitly. To find the trait child terms, we provide the function get_child_efo().

## 3 Conclusion

We have developed the first R client to the GWAS Catalog REST API, thus greatly facilitating programmatic access to the database. The main features of *gwasrapidd* are: (i) abstracting away the REST API informatic details by providing a simple and consistent interface, and (ii) a tidy data representation of the GWAS entities, i.e. of *studies*, *associations*, *variants* and *traits* in the form of in-memory relational databases. This improves data mining from within R, accelerating the integration of GWAS data into further genomic and biomedical/clinical studies.

## Supplementary Material

btz605_Supplementary_Data
