# Supplementary material for: gwasrapidd: an R package to query, download and wrangle GWAS catalog data
Source: Bioinformatics. 2019 Aug 2;36(2):649–50. doi: 10.1093/bioinformatics/btz605 (PMC9883700; doi:10.1093/bioinformatics/btz605)
Supplement: btz605_Supplementary_Data [file btz605_supplementary_data.zip › btz605-Suppl_data/Supplementary_Data1.pdf]

Search criterion	Examples	Studies	Associations	Variants	Traits
Study identifier	E P Studies: /studies/GCST001085/<br>E P Associations: /studies/GCST001085/associations<br>E P Variants: /studies/GCST001085/snps<br>E P Traits: /studies/GCST001085/efoTraits	■	■	■	■
Association identifier	E P Studies: /associations/24299710/study<br>E P Associations: /associations/24299710/<br>E P Variants: /associations/24299710/snps<br>E P Traits: /associations/24299710/efoTraits	■	■	■	■
Variant identifier	E P Studies: /singleNucleotidePolymorphisms/rs3798440/studies<br>E P Associations: /singleNucleotidePolymorphisms/rs3798440/associations<br>E P Variants: /singleNucleotidePolymorphisms/rs3798440/<br>Traits: NA	■	■	■	□
EFO trait identifier	E P Studies: /efoTraits/EFO_0000537/studies<br>E P Associations: /efoTraits/EFO_0000537/associations<br>Variants: NA<br>E P Traits: /efoTraits/EFO_0000537/	■	■	□	■
PubMed identifier	E P Studies: /studies/search/findByPublicationIdPubmedId?pubmedId=21626137<br>E P Associations: /associations/search/findByPubmedId?pubmedId=21626137<br>E P Variants: /singleNucleotidePolymorphisms/search/findByPubmedId?pubmedId=21626137<br>E P Traits: /efoTraits/search/findByPubmedId?pubmedId=21626137	■	■	■	■
Whether studies were requested by users of the GWAS Catalog or not	E P Studies: /studies/search/findByUserRequested?userRequested=false<br>Associations: NA<br>Variants: NA<br>Traits: NA	■	□	□	□
Whether studies have full summary statistics or not	E P Studies: /studies/search/findByFullPvalueSet?fullPvalueSet=true<br>Associations: NA<br>Variants: NA<br>Traits: NA	■	□	□	□
EFO URI	E P Studies: /studies/search/findByEfoUri?uri=http://www.ebi.ac.uk/efo/EFO_0004761<br>Associations: NA<br>Variants: NA<br>E P Traits: /efoTraits/search/findByEfoUri?uri=http://www.ebi.ac.uk/efo/EFO_0004761	■	□	□	■
Variant and study identifiers concomitantly	Studies: NA<br>E P Associations: /associations/search/findByRsIdAndAccessionId?rsId=rs16956936&accessionId=GCST000392<br>Variants: NA<br>Traits: NA	□	■	□	□
Genomic range (chromosome, and, start and end positions)	Studies: NA<br>Associations: NA<br>E P Variants: /singleNucleotidePolymorphisms/search/findByChromBpLocationRange?chrom=22&bpStart=1&bpEnd=15500000<br>Traits: NA	□	□	■	□
Gene name	Studies: NA<br>Associations: NA<br>E P Variants: /singleNucleotidePolymorphisms/search/findByGene?geneName=BRCA1<br>Traits: NA	□	□	■	□
EFO trait	E P Studies: /studies/search/findByEfoTrait?efoTrait=lung%20adenocarcinoma<br>E P Associations: /associations/search/findByEfoTrait?efoTrait=lung%20adenocarcinoma<br>E P Variants: /singleNucleotidePolymorphisms/search/findByEfoTrait?efoTrait=lung%20adenocarcinoma<br>E P Traits: /efoTraits/search/findByEfoTrait?trait=lung%20adenocarcinoma	■	■	■	■
Authors' reported trait	E P Studies: /studies/search/findByDiseaseTrait?diseaseTrait=Breast%20cancer<br>Associations: NA<br>E P Variants: /singleNucleotidePolymorphisms/search/findByDiseaseTrait?diseaseTrait=Breast%20cancer<br>Traits: NA	■	□	■	□

Table S1 | GWAS Catalog REST API URL endpoints. A JSON response can be embedded (E) or not (E), and paginated (P) or not (P). Full squares indicate that GWAS Catalog entities are retrievable by the search criterion, whereas outlined squares indicate otherwise. URL endpoints are shortened, i.e., the common prefix `https://www.ebi.ac.uk/gwas/rest/api/` is omitted for clarity.
