# Supplementary material for: gwasrapidd: an R package to query, download and wrangle GWAS catalog data
Source: Bioinformatics. 2019 Aug 2;36(2):649–50. doi: 10.1093/bioinformatics/btz605 (PMC9883700; doi:10.1093/bioinformatics/btz605)
Supplement: btz605_Supplementary_Data [file btz605_supplementary_data.zip › btz605-Suppl_data/Supplementary_Data2.pdf]

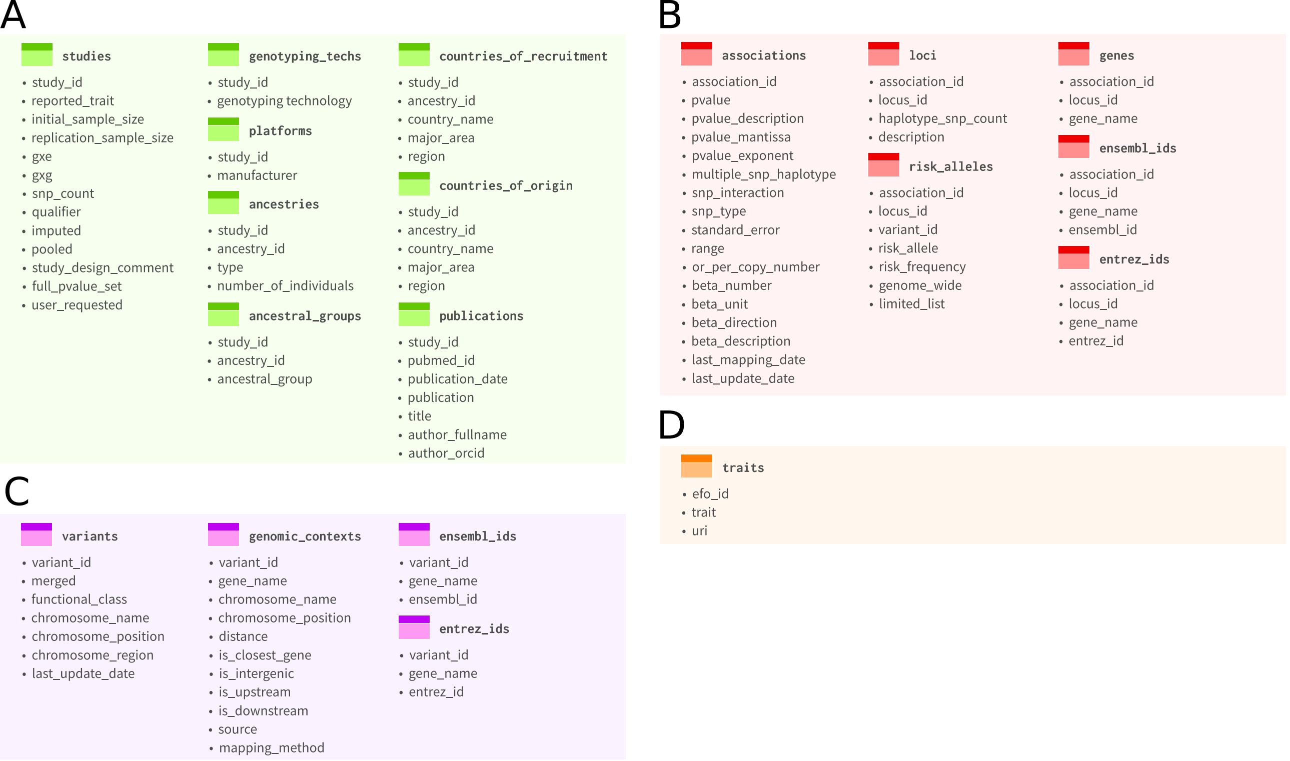

Figure S1 | S4 representation of studies, associations, variants and traits. **(A)** S4 studies object, comprising 8 slots, i.e., 8 tables: `studies`, `genotyping_techs`, `platforms`, `ancestries`, `ancestral_groups`, `countries_of_recruitment`, `countries_of_origin` and `publications`. All tables share the primary key `study_id`. **(B)** S4 associations object, comprising 6 slots, i.e., 6 tables: `associations`, `loci`, `risk_alleles`, `genes`, `ensembl_ids` and `entrez_ids`. All tables share the primary key `association_id`. **(C)** S4 variants object, comprising 4 slots, i.e., 4 tables: `variants`, `genomic_contexts`, `ensembl_ids` and `entrez_ids`. All tables share the primary key `variant_id`. **(D)** S4 traits object, with one slot, i.e., one single tables: `traits`. The traits has the primary key `efo_id`.
