# Supplementary material for: gwasrapidd: an R package to query, download and wrangle GWAS catalog data
Source: Bioinformatics. 2019 Aug 2;36(2):649–50. doi: 10.1093/bioinformatics/btz605 (PMC9883700; doi:10.1093/bioinformatics/btz605)
Supplement: btz605_Supplementary_Data [file btz605_supplementary_data.zip › btz605-Suppl_data/Supplementary_Data3.pdf]

gwasrapidd: an R package to query, download and wrangle GWAS Catalog data

Ramiro Magno and Ana-Teresa Maia *

May 2019

Case Study

To illustrate how to use *gwasrapidd*, we take as a motivating example the work by Light *et al.* (2014). In this work, the authors aimed at characterising chromatin status at allelic resolution, as a strategy for elucidanting the cis-regulatory mechanisms behind complex disease risk. To perform this characterisation, the authors started by selecting variants previously reported in the GWAS Catalog for autoimmune disease. We enact now what could have been these first steps of their approach using *gwasrapidd*. We start by loading *gwasrapidd*:

```
library(gwasrapidd)
```

Then, we query the GWAS Catalog for *studies* by searching by *autoimmune disease* (an Experimental Factor Ontology (EFO) trait):

```
my_studies <-
  get_studies(efo_trait = 'autoimmune disease')
```

One can now check how many GWAS studies were retrieved using the function `n()`. The same function could be used for the other entities: `associations`, `variants` or `traits`.

```
n(my_studies)
#> [1] 1
my_studies@studies$study_id
#> [1] "GCST003097"
```

Seemingly, only one study matched exactly `'autoimmune disease'`: the study with the identifier `GCST003097`. We can inspect the original publication(s) that underlie this GWA study entry in the Catalog. For example, to access the associated publication title one can access the `title` variable from the `publications` table:

```
my_studies@publications$title
#> [1] "Meta-analysis of shared genetic architecture across ten pediatric autoimmune diseases."
```

To quickly browse to the PubMed entry for this publication, the user may use the helper function `open_in_pubmed()`:

```
# This launches your web browser at https://www.ncbi.nlm.nih.gov/pubmed/26301688
open_in_pubmed(my_studies@publications$pubmed_id)
```

Now if we want to know the variants previously associated with autoimmune disease, as used by Light *et al.* (2014), we need to retrieve statistical association information on these variants, and then apply a filter using the same level of significance $P < 1\times10^{-6}$ (Light *et al.*, 2014). A quick inspection at the *gwasrapidd* cheatsheet (Additional file 2: gwasrapidd cheatsheet) hints that statitiscal information, such as p-values and odds ratios can be found in the `associations` table of the `associations` S4 class object. So, we can get the associations by the previously found *study* identifier (`GCST003097`):

```r
# Alternative query that would work too:
# get_associations(
#   efo_trait = 'autoimmune disease'
#   )
my_associations <- get_associations(study_id = "GCST003097")
```

We find 46 *associations*:

```r
n(my_associations)
#> [1] 46
```

However, it might be that not all variants meet the level of significance, as required by Light *et al.* (2014). We use now functions from the *dplyr* package (Wickham *et al.*, 2019) to filter the table `associations` from the S4 object `associations` based on the p-value (`pvalue` column).

```r
# Get association ids
# for which pvalue is less than 1e-6.
dplyr::filter(
  my_associations@associations,
  pvalue < 1e-6) %>%
  dplyr::pull(association_id) ->
  association_ids
```

Having the *association* identifiers (`association_ids`) that meet the requirement $P < 1 \times 10^{-6}$, we can easily create a new S4 object (`my_associations2`) containing only those *associations* using the subset operator `` `[` ``:

```r
# Extract associations by association id
my_associations2 <- my_associations[association_ids]
```

The subset operator `` `[` `` can also be used with `integer` values to subset by position. Note that both ways of subsetting will filter all tables within the S4 object for the matched identifiers. Now we can check how many associations are still present in `my_associations2`:

```r
n(my_associations2)
#> [1] 44
```

Of the 46 associations found in GWAS Catalog, 44 meet the p-value threshold of 1×10^{-6}. Finally, to see variants, we just need to access the table `risk_alleles` from the `my_associations2` object. Here are the variant identifers, risk alleles and risk frequency:

```r
my_associations2@risk_alleles[c('variant_id', 'risk_allele', 'risk_frequency')]
#> # A tibble: 44 x 3
#>    variant_id  risk_allele risk_frequency
#>    <chr>       <chr>                <dbl>
#>  1 rs2066363   C                     0.34
#>  2 rs114846446 A                     0.01
#>  3 rs7672495   C                     0.18
...
```

References

Light, N., *et al.* (2014). Interrogation of allelic chromatin states in human cells by high-density chip-genotyping. *Epigenetics*, **9**(9), 1238–1251.

Wickham, H., *et al.* (2019). *dplyr: A Grammar of Data Manipulation*. R package version 0.8.0.1.
